# Actionability of HER2-amplified circulating tumor cells in HER2-negative metastatic breast cancer: the CirCe T-DM1 trial

**DOI:** 10.1186/s13058-019-1215-z

**Published:** 2019-11-14

**Authors:** William Jacot, Paul Cottu, Frederique Berger, Coraline Dubot, Laurence Venat-Bouvet, Alain Lortholary, Hugues Bourgeois, Marc Bollet, Veronique Servent, Elisabeth Luporsi, Marc Espié, Severine Guiu, Veronique D’Hondt, Veronique Dieras, Marie-Paule Sablin, Etienne Brain, Souhir Neffati, Jean-Yves Pierga, Francois-Clement Bidard

**Affiliations:** 1 Department of Medical Oncology, Institut du Cancer de Montpellier, Montpellier, France; 2 Institut de Recherche en Cancérologie de Montpellier (IRCM), Inserm U1194, Université de Montpellier, Institut Régional du Cancer de Montpellier (ICM), Montpellier, France; 3 Montpellier University, Montpellier, France; 4 Department of Medical Oncology, Institut Curie, PSL Research University, Saint Cloud, France; 5 Biometry and Clinical Trial Promotion Units, Institut Curie, PSL Research University, Saint Cloud, France; 6 Department of Medical Oncology, Centre Hospitalier Universitaire, Limoges, France; 7 Department of Medical Oncology, Centre Catherine de Sienne, Nantes, France; 8 Department of Medical Oncology, Clinique Victor Hugo, Le Mans, France; 9 Department of Radiation Therapy, Clinique Hartmann, Neuilly, France; 10 Department of Medical Oncology, Centre Oscar Lambret, Lille, France; 11 Department of Medical Oncology, Institut de Cancérologie de Lorraine, Nancy, France; 12 Department of Medical Oncology, Hôpital Saint Louis, Paris, France; 13 Université Paris Descartes, Paris, France; 14 Laboratory of Circulating Tumor Biomarkers, Institut Curie, PSL Research University, Paris, France; 15 UVSQ, Paris Saclay University, Saint Cloud, France

**Keywords:** Circulating tumor cells, Metastatic breast cancer, HER2, Trastuzumab-emtansine, Liquid biopsy

## Abstract

**Background:**

In this prospective phase 2 trial, we assessed the efficacy of trastuzumab-emtansine (T-DM1) in HER2-negative metastatic breast cancer (MBC) patients with HER2-positive CTC.

**Methods:**

Main inclusion criteria for screening were as follows: women with HER2-negative MBC treated with ≥ 2 prior lines of chemotherapy and measurable disease. CTC with a *HER2*/CEP17 ratio of ≥ 2.2 by fluorescent in situ hybridization (CellSearch) were considered to be *HER2*-amplified (*HER2*_*amp*_). Patients with ≥ 1 *HER2*_*amp*_ CTC were eligible for the treatment phase (T-DM1 monotherapy). The primary endpoint was the overall response rate.

**Results:**

In 154 screened patients, ≥ 1 and ≥ 5 CTC/7.5 ml of blood were detected in *N* = 118 (78.7%) and *N* = 86 (57.3%) patients, respectively. ≥1 *HER2*_*amp*_ CTC was found in 14 patients (9.1% of patients with ≥ 1 CTC/7.5 ml). Among 11 patients treated with T-DM1, one achieved a confirmed partial response. Four patients had a stable disease as best response. Median PFS was 4.8 months while median OS was 9.5 months.

**Conclusions:**

CTC with *HER2* amplification can be detected in a limited subset of HER2-negative MBC patients. Treatment with T-DM1 achieved a partial response in only one patient.

**Trial registration:**

NCT01975142, Registered 03 November 2013

## Introduction

In view of the significant efficacy of anti-HER2 targeted therapies on HER2-positive breast cancers, assessment of HER2 status has become a cornerstone of the current breast cancer management. HER2 testing on tumor tissue has been standardized by successive guidelines and relies first on immunohistostaining [1–3]. In ambiguous cases, in situ hybridization (ISH) assays remain the gold standard as it directly assesses *HER2* gene copy number and any chromosome 17 polysomy. As *HER2* amplification is an early oncogenic event, HER2 status has been found to be different between primary tumors and matched metastatic tissue in fewer than 10% of patients [4,5]. When clinically feasible, HER2 status should therefore be reassessed on metastatic tissue sample in metastatic breast cancer (MBC) patients [6].

While invasive biopsy of a metastatic lesion may not always be feasible or contributive, circulating tumor biomarkers promise to become a noninvasive surrogate for tissue-based biomarkers, including HER2 status [7,8]. Many detection platforms have demonstrated that HER2 immunocytostaining and ISH techniques can be applied to circulating tumor cells (CTC) [9–12]. Some reports have also suggested a significant heterogeneity between the HER2 status of primary breast tumors and that of matched CTC sampled during the course of metastatic disease [9,13–21].

Within the current armamentarium of HER2-targeting drugs, trastuzumab-emtansine (T-DM1) has demonstrated its efficacy in the metastatic setting, starting from the second line of therapy [22,23]. This antibody-drug conjugate is given as a single agent and therefore represents an exquisite targeting of HER2-positive tumor cells, with no direct action on HER2-negative tumor cells.

The purpose of the phase 2 “CirCe T-DM1” trial was to investigate the clinical actionability of CTC-based HER2 status assessment. In a screening step, HER2-negative MBC patients were screened for HER2-positive CTC with the most reliable technical approach, ISH. During the treatment step, HER2-negative MBC patients with *HER2*-amplified (*HER2*_*amp*_) CTC were treated with T-DM1 given as a single agent. Final results of the screening and the treatment steps are reported here.

## Materials and methods

The CirCe T-DM1 trial was approved by the regional ethics committee (*CPP Ile de France I*) and has been registered (EudraCT 2012-005155-16; NCT01975142). All patients provided written informed consent at inclusion both in the screening step and in the treatment step.

### Screening

Main inclusion criteria for the screening step were as follows: women with HER2-negative adenocarcinoma of the breast, as assessed by immunohistostaining or/and ISH on the primary breast tumor (HER2 status reassessment on metastatic lesions was not mandatory but, when performed, had to be HER2-negative); metastatic and/or inoperable locoregional relapse progressing on at least two prior lines of systemic chemotherapy; measurable disease (RECIST v1.1); WHO performance status of 0–2; adequate laboratory parameters; and cardiac function.

Three 7.5-ml blood samples were drawn in CellSave® tubes from patients included in the screening step. Tubes were shipped at room temperature to a central laboratory (Janssen Diagnostics), located in Beerse, Belgium. The fluorescent-ISH (FISH) analysis was a multistep process, performed by Janssen Diagnostics under blinded conditions. CTC were first detected and located by immunocytofluorescence (standard CellSearch® technique); the slide was then submitted to FISH and screened a second time for fluorescent signal. Results were available within 7 days and included the following: number of CTC detected, absolute numbers of *HER2* copies and chromosome 17 centromeres (CEP17) for each CTC with interpretable FISH assay, at the single cell level, and the results of internal negative controls (i.e. *HER2* and CEP17 signals observed in leukocytes from the same sample). CTC displaying a *HER2*/CEP17 ratio of ≥ 2.2 (as per the 2007 ASCO/CAP guidelines [1]) and/or > 6 HER2 copies were considered as *HER2*_*amp*_. CTC with high numbers of *HER2* copies without CEP17 signal available or with a *HER2*/CEP17 ratio < 2.2 were not considered to be *HER2*_*amp*_. Patients with no CTC or with non-*HER2*_*amp*_ CTC were then considered to be off-study.

### Treatment and assessment

Patients with ≥ 1 *HER2*_*amp*_ CTC detected at the screening step were eligible for the treatment step, which consisted of T-DM1 monotherapy at the standard dose of 3.6 mg/kg IV every 3 weeks (dose reductions were allowed in the case of toxicity). Clinical and laboratory examinations were performed at each cycle, and radiological evaluation was performed every 6 weeks, according to RECIST 1.1. A second CTC count with *HER2* FISH was performed after 1 cycle of therapy, but clinicians were blinded to the results.

### Statistics

We hypothesized that the efficacy of T-DM1 may differ according to the absolute number of *HER2*_*amp*_ CTC detected at the screening step; also, a very low HER2_amp_ CTC count might be distributed by Poisson’s law of rare event and therefore turn out to be less reproducible. We therefore distinguished two populations of treated patients: *HER2*_*amp*_ CTC_low_ and *HER2*_*amp*_ CTC_high_ populations, corresponding to patients with 1–2 *HER2*_*amp*_ or ≥ 3 *HER2*_*amp*_ CTC detected, respectively.

The primary objective of this study was to report the efficacy of T-DM1 in the two populations. The primary endpoint was the objective response rate among treated patients. Secondary objectives included progression-free survival (PFS; defined as the time between inclusion in the treatment step and tumor progression or death, whichever came first), overall survival (OS), duration of response, and biomarker responses.

The design of this multicenter phase 2 trial was derived from a multiple-stage Fleming design [24,25], the two populations (*HER2*_*amp*_ CTC_low_ and *HER2*_*amp*_ CTC_high_) being assessed as separate cohorts. In a first stage, seven patients had to be included in each of the two cohorts (*N* = 7 *HER2*_*amp*_ CTC_low_ and *N* = 7 *HER2*_*amp*_ CTC_high_). T-DM1 was estimated to be effective when it yielded a response rate of 25% (H1) and ineffective when it yielded a response rate < 5% (H0). After inclusion of seven patients, the study could be stopped in the corresponding population for inefficacy (no response observed) or efficacy (three or more response observed). When one to two responses were observed, another 7 patients had to be included in each cohort before drawing conclusions. In total, with an anticipated 10% detection rate of HER2_amp_ CTC, about 280 patients could have been included in the screening step. With the abovementioned H0 and H1 hypotheses, this trial had an overall alpha risk of 0.06 and a power of 0.94. Data are available upon request.

## Results

### Detection of HER2_amp_ CTC

**Fig. 1:**
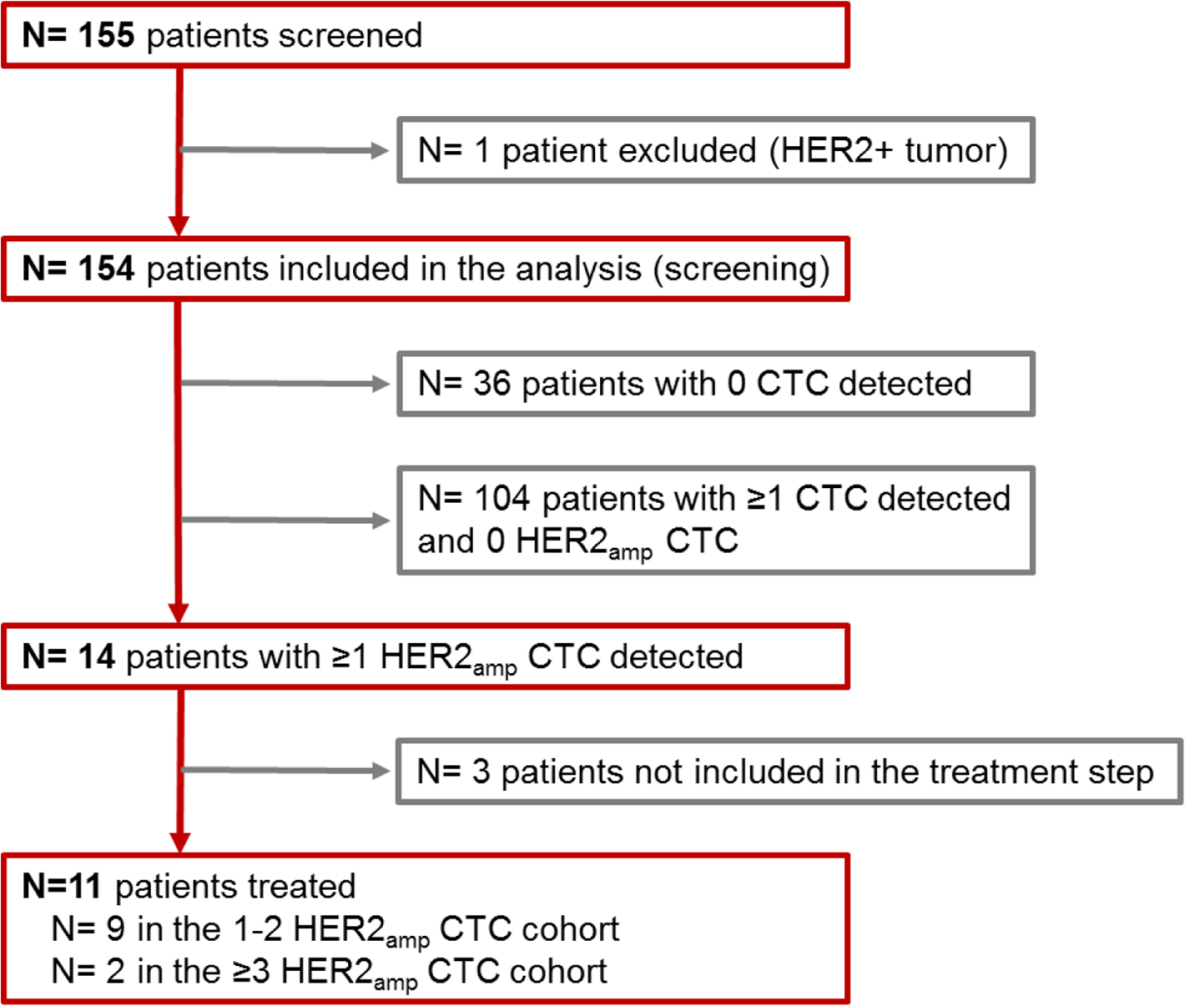
Flow chart

This study was open to accrual from 11/2013 to 09/2016 in 10 centers. One hundred fifty-five patients were included in the screening phase, and one patient was subsequently excluded due to HER2 positivity on metastasis. Patient characteristics are shown in Table 1. Among 154 included patients, 80 (51.9%) patients underwent a biopsy of their local/distant relapse which confirmed the HER2-negative tumor status; 87 (57.2%) patients received 3 or more lines of chemotherapy for their metastatic disease. The study flow chart is displayed in Fig. 1.

**Table 1:** Characteristics of screened patients and *HER2*_amp_ CTC detection

Patients characteristics	Included pts. (*N* = 154) *N* (%)	Pts. with ≥1CTC (*N* = 118) *N* (%)	Pts. with ≥1CTC and interpretable FISH^c^ (*N* =79) *N* (%)	Pts. with ≥ 1 HER2_amp_ CTC (*N* = 14) *N* (%)
Age at inclusion				
≤ 50 years	26 (17.0%)	18 (15.4%)	12 (15.2%)	3 (21.4%)
> 50 years	127 (83.0%)	99 (84.6%)	67 (84.8%)	11 (78.6%)
NA	1	1		
Performance status				
PS 0	63 (44.4%)	40 (37.4%)	25 (33.8%)	3 (21.4%)
PS 1	78 (54.9%)	66 (61.7%)	48 (64.9%)	10 (71.4%)
PS 2	1 (0.7%)	1 (0.9%)	1 (1.4%)	1 (7.1%)
NA	12	11	5	
Tumor type				
NST	135 (89.4%)	103 (88.8%)	67 (85.9%)	13 (92.9%)
Lobular	13 (8.6%)	10 (8.6%)	10 (12.8%)	1 (7.1%)
Other	3 (2.0%)	3 (2.6%)	1 (1.3%)	0 (0%)
NA	3	2	1	
Tumor grade				
Grade I	10 (7.4%)	9 (8.7%)	7 (9.9%)	2 (16.7%)
Grade II	80 (59.3%)	60 (58.3%)	44 (62.0%)	6 (50.0%)
Grade III	45 (33.3%)	34 (33.0%)	20 (28.2%)	4 (33.3%)
NA	19	15	8	2
Receptor status on primary tumor ^a^				
ER− PR− HER2−	15 (10.1%)	10 (8.9%)	6 (8.1%)	0 (0%)
ER+ and/or PR+, HER2−	125 (84.5%)	98 (87.5%)	67 (90.5%)	12 (100%)
Not done	8 (5.4%)	4 (3.6%)	1 (1.4%)	0 (0%)
NA	6	6	5	2
Receptor status on local/distant relapse^a^				
ER− PR− HER2−	17 (19.3%)	10 (15.4%)	8 (19.5%)	
ER+ and/or PR+, HER2−	63 (71.6%)	49 (75.4%)	30 (73.2%)	7 (87.5%)
Not done	8 (9.1%)	6 (9.2%)	3 (7.3%)	1 (12.5%)
NA	66	53	38	6
Number of prior lines of chemotherapy^b^				
2	65 (42.8%)	46 (39.7%)	33 (42.3%)	3 (21.4%)
3	42 (27.6%)	34 (29.3%)	20 (25.6%)	4 (28.6%)
≥ 4	45 (29.6%)	36 (31.0%)	25 (32.1%)	7 (50.0%)
NA	2	2	1	
Number of CTC detected				
Screening CTC = 0	32 (21.3%)			
Screening CTC [1–4]	32 (21.3%)	32 (27.1%)	11 (13.9%)	0 (0%)
Screening CTC ≥ 5	86 (57.3%)	86 (72.9%)	68 (86.1%)	14 (100%)
NA	4			

*NST* invasive carcinoma of no special type, *ER* estrogen receptor, *PR* progesterone receptor

^a^One patient with a history of HER2-positive primary tumor was excluded and is not shown in this table

^b^Chemotherapies administered for metastatic disease

^c^Interpretable FISH results: at least 1 CTC with both *HER2* and CEP17 signals observed, with negative internal controls

Among the 154 patients screened, ≥ 1 and ≥ 5 CTC/7.5 ml of blood were detected in *N* = 118 (78.7%) and *N* = 86 (57.3%) patients, respectively. Sixty-seven of the 79 patients (84.8%) of patients with a performance status of 1 or 2 had ≥ 1 CTC/7.5 ml detected, versus 40/63 (63.4%) patients with a performance status of 0 (*p* = 0.006).

**Fig. 2:**
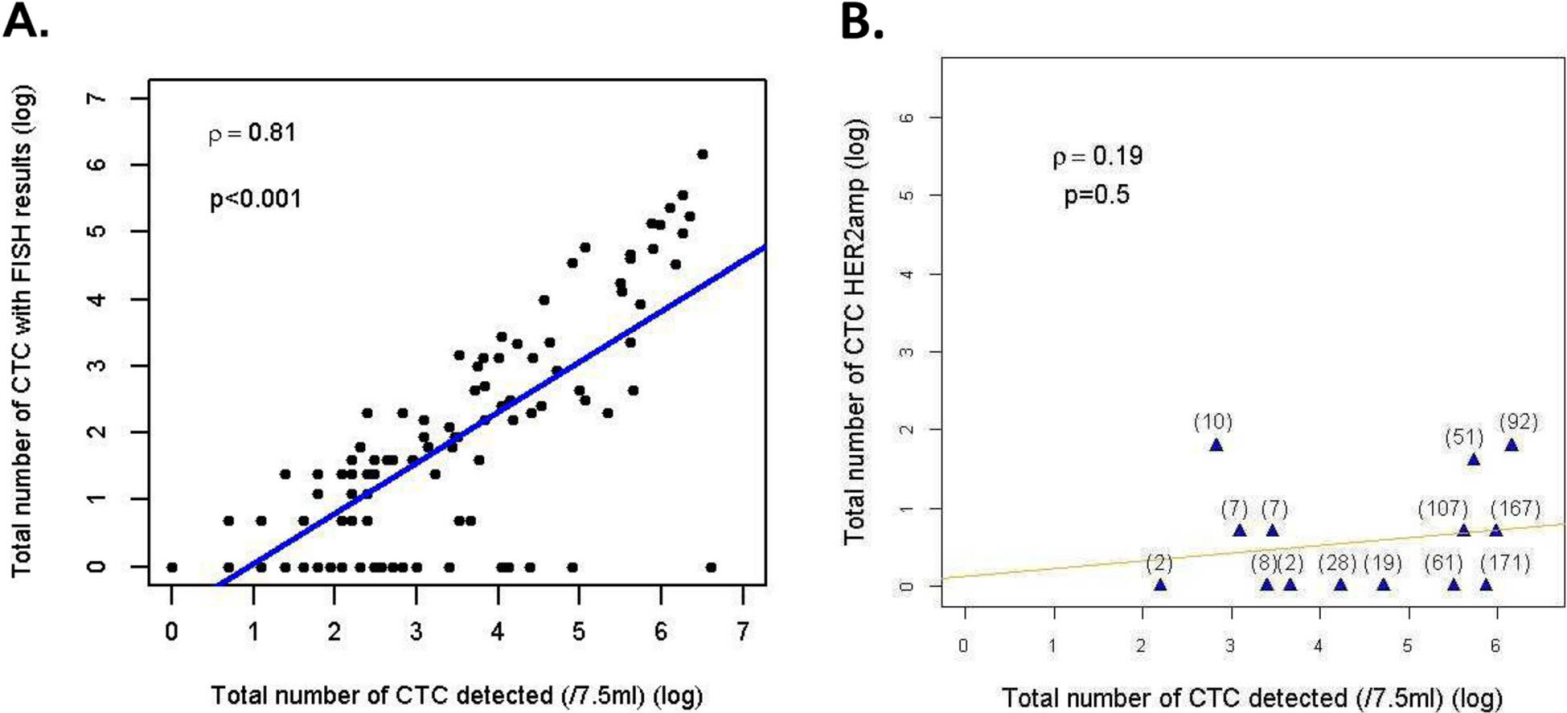
Correlations between absolute CTC count and downstream CTC characterization. **a** Correlation between the number of CTC detected and the number of CTC with interpretable FISH results. **b** Correlation, in the 14 patients with ≥ 1 HER2_amp_ CTC at the screening step, between the number of (i) CTC, (ii) CTC with interpretable FISH result, and (iii) HER2_amp_ CTC. **b** Correlation between the number of CTC detected and the number of JER2^amp^ CTC for the 14 patients with ≥ 1 HER^amp^ CTC detected. For each patient, the number of CTC with interpretable FISH results is shown in brackets.

FISH analysis was performed in samples with ≥ 1 CTC. For any given CTC, FISH results were considered to be interpretable when both *HER2* and CEP17 signals could be assessed on at least 1 CTC and when leukocytes from the same sample showed normal *HER2* and CEP17 signals. Overall, among the 7124 CTC detected by the CellSearch system in 118 patients with ≥ 1 CTC/7.5 ml at the screening stage, FISH was deemed interpretable for 1652 CTC in 79 patients (66.9% of patients with ≥ 1 CTC /7.5 ml). As expected, the probability of having ≥ 1 CTC on a blood sample with an interpretable FISH result was correlated with the total number of CTC detected (Table 1; Fig. 2a, *ρ* = 0.81, *p* = < 0.001).

Overall, ≥ 1 *HER2*_*amp*_ CTC was found in 14 patients (9.1%). The detection of *HER2*_*amp*_ CTC was not correlated to the total number of detected CTC (Fig. 2b) but was associated with performance status (*p* = 0.02). Among patients with ≥ 1 *HER2*_*amp*_ CTC, absolute CTC count, CTC with interpretable FISH, and CTC with or without *HER2amp* are displayed in Table 2. Seven patients (50%) had 1 *HER2*_*amp*_ CTC. Among patients with ≥ 2 CTC with interpretable FISH results, the median ratio of *HER2*_*amp*_ CTC among all CTC with interpretable FISH results was 8.2% (range 0.6–60%).

**Table 2:** Details of HER2_amp_ CTC detection and characteristics (*) HER2/CEP17 number and ratio in the 17 detected *HER2*_amp_ CTC were as follows: 3/1/3.0 for each of the 17 CTC.

Patient	Step	No. of CTC detected (/7.5 ml)	No. of CTC with FISH	No. of *HER2*_amp_ CTC	HER2 and CEP17 for each *HER2*_amp_ CTC		
					*N* CEP17	*N* HER2	HER2/CEP17 ratio
Patients screened with *HER2*_amp_ CTC, with no further sample (not treated or not assessed)							
03-020-1	Screening	9	2	1	1	3	3.0
03-030-1	Screening	247	61	1	1	3	3.0
03-031-1	Screening	69	28	1	2	6	3.0
03-039-1	Screening	111	19	1	1	7	7.0
04-003-1	Screening	30	8	1	2	6	3.0
10-004-1	Screening	39	2	1	2	5	2.5
03-037-2	Screening	308	51	5	2	6	3.0
					2	6	3.0
					2	5	2.5
					2	5	2.5
					2	5	2.5
10-005-2	Screening	475	92	6	1	10	10.0
					1	10	10.0
					1	8	8.0
					1	3	3.0
					2	5	2.5
					2	5	2.5
Patients screened with *HER2*_amp_ CTC with sample during therapy							
01-017-1	Screening	22	7	2	1	3	3.0
					1	3	3.0
	Cycle #2	9	5	0			
	Progression	22	9	0			
05-005-1	Screening	32	7	2	1	3	3.0
					1	3	3.0
	Cycle #2	10	6	0			
05-006-2	Screening	17	10	6	1	3	3.0
					1	3	3.0
					1	3	3.0
					1	3	3.0
					1	3	3.0
					1	3	3.0
	Cycle #2	57	31	17	(*) see caption		
07-010-1	Screening	397	167	2	1	5	5.0
					2	8	4.0
	Cycle #2	445	216	3	2	10	5.0
					3	10	3.3
					1	3	3.0
08-001-1	Screening	355	171	1	1	3	3.0
	Cycle #2	136	95	3	1	6	6.0
					2	6	3.0
					1	3	3.0
	Progression	665	484	4	1	3	3.0
					1	3	3.0
					1	3	3.0
					2	5	2.5
08-002-1	Screening	275	107	2	1	4	4.0
					2	5	2.5
	Cycle #2	242	70	5	1	4	4.0
					1	3	3.0
					1	3	3.0
					1	3	3.0
					1	3	3.0
					1	3	3.0

In September 2016, the supplier of the FISH CTC assay, blinded to the ongoing study results, discontinued development of this test. Patient screening and enrollment was therefore stopped prior to completion of the targeted study enrollment.

### Patient outcomes

In the overall screening population, CTC count confirmed its prognostic value on PFS and OS in univariate and multivariate analyses (Additional file 1: Figure S1; Additional file 2: Table S1).

In patients with no CTC detected at screening, observed median PFS and OS were 11.6 months (95%CI = [7.3;17.5]) and 31 months (95%CI = [25;not reached]), respectively. In patients with ≥ 1 CTC but no *HER2*_*amp*_ CTC detected at screening, observed median PFS and OS were 5.6 months (95%CI = [4.1;7.4]) and 10.7 months (95%CI = [8.1;14.8]), respectively.

Eleven patients with ≥ 1 *HER2*_*amp*_ CTC detected at screening have been included in the treatment step: 9 patients in the *HER2*_*amp*_ CTC_low_ cohort (1 or 2 *HER2*_*amp*_ CTC detected) and 2 patients in the *HER2*_*amp*_ CTC_high_ cohort (≥3 CTC *HER2*_*amp*_ CTC detected); all patients included in the treatment step received at least one dose of T-DM1.

**Fig. 3:**
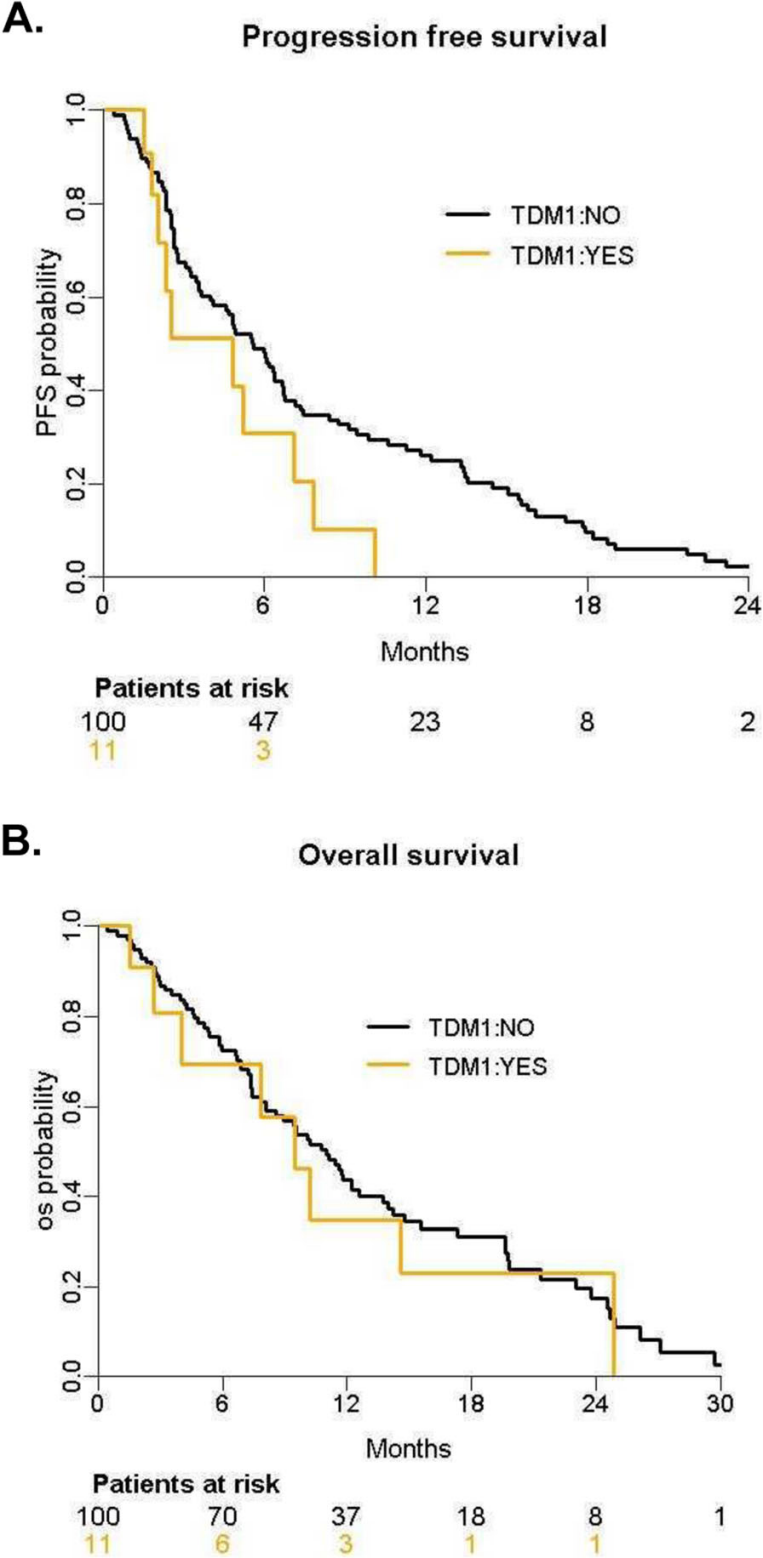
Survival curves**.** Progression-free survival (**a**) and overall survival (**b**) for patients with ≥ 1 HER2_amp_ CTC treated with T-DM1 (in yellow) and patients with ≥ 1 CTC but no HER2_amp_ CTC, treated per standard of care (in black)

Overall, one of the first seven patients (patient #03-020-1 in Table 2) included in the *HER2*_*amp*_ CTC_low_ cohort achieved a confirmed partial response, which allowed to enroll two more patients in that cohort prior to the study discontinuation. The overall objective response rate was therefore 11.1% (95%CI = [0.3; 48.3]) in the CTC_low_ cohort (*N* = 9 patients) and 9.1% (95%CI = [0.23;41.3]) in all treated patients (*N* = 11 patients). The duration of this partial response was 7.1 months. At time of progression, this patient underwent a biopsy of a metastasis that responded to T-DM1, which confirmed the lack of HER2 amplification. Four patients had a stable disease as the best response. Another patient in the same cohort displayed a drop of serum tumor markers (CA15-3 and CEA) after 1 cycle of T-DM1 but was withdrawn from the study thereafter due to a grade III pneumonitis. No other toxicities potentially related to T-DM1 have been observed. Overall, in the 11 patients with *HER2*_*amp*_ CTC, observed median PFS and OS were 4.8 months 95%CI = [2;not reached] and 9.5 months 95%CI = [4.0;not reached], respectively **(**Fig. 3**)**.

Table 2 shows the total CTC count and *HER2*_amp_ CTC count during therapy for the 11 treated patients, whenever available.

## Discussion

This study exemplifies the promises and the pitfalls of using a “liquid biopsy” to guide patient towards a personalized therapy.

Firstly, each biomarker (such as *HER2* amplification in our study) of interest on CTC requires the detection of at least one CTC. In a prior study, we reported an overall good concordance between the HER2 status of the primary breast cancer and that of CTC (assessed by immunocytofluorescence), only if a sufficient number of CTC was assessed [26]. Our results show that the feasibility of *HER2* FISH on CTC is directly correlated with the number of CTC detected, which is per se an independent prognostic factor, as demonstrated elsewhere [27] and confirmed in our study. The limited number of CTC with interpretable FISH results was maybe related to the two-step process, some cells detaching from the slide after the CTC count, during the FISH procedure.

Secondly, we found out—using ISH, the most reliable assay for HER2 status—that nearly 10% of MBC patients with HER2-negative tumor exhibit *HER2*_*amp*_ CTC. The presence of such HER2-positive CTC (detected by ISH or immunocytology) has been reported by several observational studies [9,13–19]. In our study, both the absolute number of *HER2*_*amp*_ CTC and the *HER2*_*amp*_/HER2-negative CTC ratio were low, suggesting that these *HER2*_*amp*_ CTC account for a minority of the tumor burden and that *HER2*_*amp*_ subclones do not expand significantly during therapy in MBC patients.

From a statistical perspective, the main limitation of our study is the low number of patients that were treated with T-DM1, so the efficacy of T-DM1 in patients with HER2-negative tumors and HER2_amp_ CTC, although unlikely, cannot formally be ruled out. In addition to the low objective response rate, the median PFS achieved by T-DM1 in patients with HER2_amp_ CTC compares unfavorably to that observed in patients with ≥ 1 CTC but no HER2_amp_ CTC.

In our study, the use of T-DM1 allowed assessing the efficacy of “pure” anti-HER2 therapy in the setting of a single-arm phase 2 study, without any other anti-tumor therapy. A different approach, investigated in the DETECT III study (NCT01619111), is to measure whether adding an anti-HER2 agent to a chemotherapy backbone would benefit to HER2-negative MBC patients with HER2-positive CTC, assessed by immunocytofluorescence (design reviewed in [28]). Results of this randomized phase III study are awaited and will complete our current understanding of the clinical actionability of *HER2*_amp_ CTC in HER2-negative MBC patients.

The lack of synchronous metastatic tissue biopsy at time of treatment initiation also prevents us from comparing the *HER2* amplification status between CTC and metastatic tissue. Recent preclinical experiments suggested that HER2-positive CTC retrieved from HER2-negative MBC patients are more proliferative but not addicted to HER2 signaling [14]. However, trastuzumab-deruxtecan, a newer trastuzumab-drug conjugate, demonstrated an efficacy in some patients with HER2-negative metastatic cancers [29]; this efficacy signal is now investigated in a phase 3 trial (NCT03734029).

## Conclusions

CTC with *HER2* amplification can be detected by ISH in about one tenth of HER2-negative MBC patients with detectable CTC. The very limited efficacy of single agent T-DM1 in that setting may be related to the fact that HER2-amplified CTC represented only a fraction of the total CTC detected in treated patients.

## Supplementary information

**Additional file 1.**  **Figure S1.** Survival by CTC count at the screening step. a: Progression-Free Survival. b: Overall Survival.

**Additional file 2.**  **Table S1.** Univariate and multivariate analyses for PFS and OS (screened population). For multivariate analyses, only significant results are shown. MBC: metastatic breast cancer.

## Data Availability

The datasets generated and/or analyzed during the current study are not publicly available due to privacy of clinical data but are available from the corresponding author on reasonable request.

## Abbreviations

CEP17: Chromosome 17 centrosome
CTC: Circulating tumor cell
FISH: Fluorescent in situ hybridization
HER2: Human EGFR-related receptor 2
HER2_amp_: Human EGFR-related receptor 2-amplified
ISH: In situ hybridization
MBC: Metastatic breast cancer
OS: Overall survival
PFS: Progression-free survival
T-DM1: Trastuzumab emtansine
