# Supplementary material for: Actionability of HER2-amplified circulating tumor cells in HER2-negative metastatic breast cancer: the CirCe T-DM1 trial
Source: Breast Cancer Res. 2019 Nov 14;21:121. doi: 10.1186/s13058-019-1215-z (PMC6854749; doi:10.1186/s13058-019-1215-z)
Supplement: Supplementary file 1 — Additional file 1. Figure S1. Survival by CTC count at the screening step. a: Progression-Free Survival. b: Overall Survival. [file 13058_2019_1215_MOESM1_ESM.docx]

**Supplementary Figure 1: survival by CTC count at the screening step**

**a: Progression-Free Survival**

**
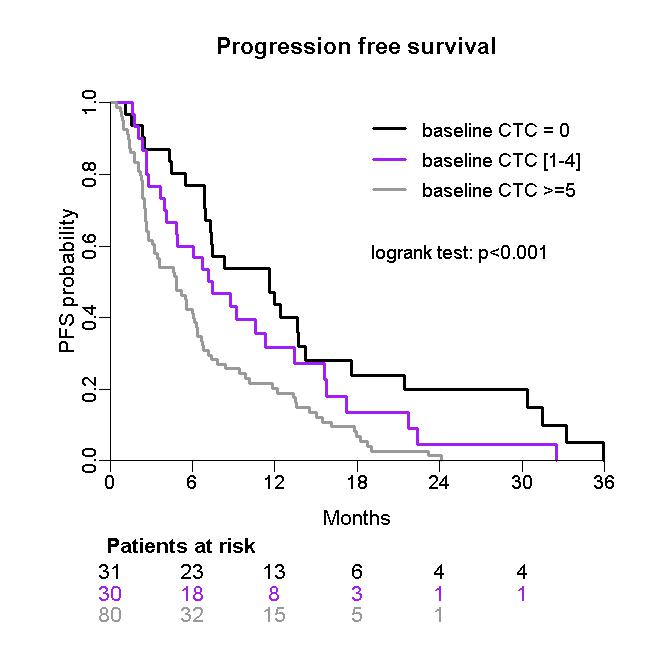
**

**b: Overall Survival**

**
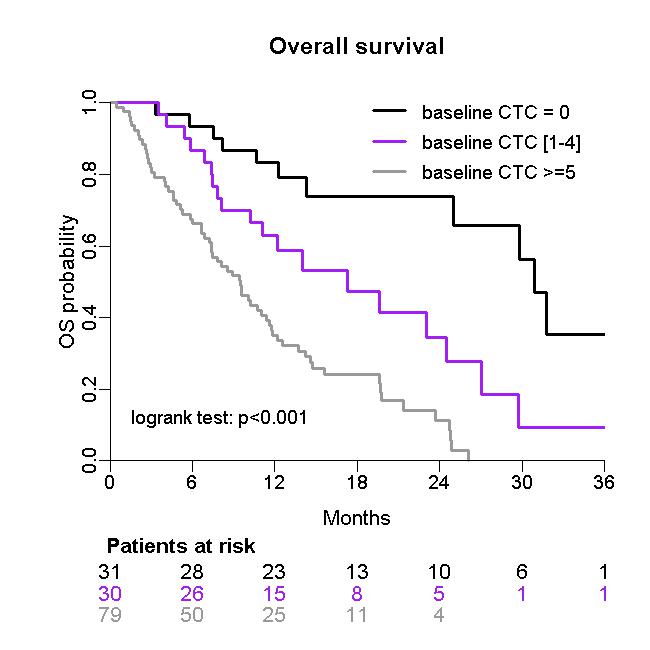
**
