# Supplementary material for: Actionability of HER2-amplified circulating tumor cells in HER2-negative metastatic breast cancer: the CirCe T-DM1 trial
Source: Breast Cancer Res. 2019 Nov 14;21:121. doi: 10.1186/s13058-019-1215-z (PMC6854749; doi:10.1186/s13058-019-1215-z)
Supplement: Supplementary file 2 — Additional file 2. Table S1. Univariate and multivariate analyses for PFS and OS (screened population). For multivariate analyses, only significant results are shown. MBC: metastatic breast cancer. [file 13058_2019_1215_MOESM2_ESM.docx]

**Supplementary Table 1.**

Univariate and multivariate analyses for PFS and OS (screened population). For multivariate analyses, only significant results are shown. MBC: metastatic breast cancer.

|  |  | **Progression-Free Survival** | | | | | | **Overall Survival** | | | | | | |
| --- | --- | --- | --- | --- | --- | --- | --- | --- | --- | --- | --- | --- | --- | --- |
|  |  | **Univariate analysis** | | | **Multivariate analysis** | | | **Univariate analysis** | | | **Multivariate analysis** | | | |
|  | N pts | HR | 95%CI | P value | HR | 95%CI | P value | HR | 95%CI | P value | HR | 95%CI | P value |  |
| **Number of prior lines of chemotherapy for MBC** |  |  |  | 0.005 |  |  | 0.009 |  |  | 0.03 |  |  | 0.04 |  |
| 2 | 65 | 1.0 |  |  | 1,0 |  |  | 1.0 |  |  | 1.0 |  |  |  |
| 3 | 42 | 1.5 | [1.0;2.2] |  | 1,4 | [0.9;2.1] |  | 1.5 | [0.9;2.4] |  | 1.5 | [0.9;2.4] |  |  |
| ≥4 | 45 | 2.0 | [1.3;3.0] |  | 2,0 | [1.3;3.0] |  | 1.8 | [1.1;3.0] |  | 1.9 | [1.2;3.1] |  |  |
| **Any prior endocrine therapy for MBC** |  |  |  | 0.09 |  |  | 0.05 |  |  | 0.99 |  |  |  |  |
| No | 56 | 1.0 |  |  | 1,0 |  |  | 1.0 |  |  |  |  |  |  |
| Yes | 96 | 0.7 | [0.5;1.1] |  | 0,7 | [0.5;1.0] |  | 1.0 | [0.7;1.5] |  |  |  |  |  |
| **CTC count**  **at screening** |  |  |  | <0.001 |  |  | <0.001 |  |  | <0.001 |  |  | <0.001 |  |
| CTC<5 | 64 | 1.0 |  |  | 1,0 |  |  | 1.0 |  |  | 1.0 |  |  |  |
| CTC≥5 | 86 | 2.0 | [1.4;2.8] |  | 1,9 | [1.3;2.7] |  | 3.6 | [2.3;5.8] |  | 3.7 | [2.3;6.0] |  |  |
| **Menopausal status**  **at screening** |  |  |  | 0.93 |  |  |  |  |  | 0.34 |  |  |  |  |
| Post-menopausal: NO | 24 | 1.0 |  |  |  |  |  | 1.0 |  |  |  |  |  |  |
| Post-menopausal: YES | 122 | 1.0 | [0.6;1.6] |  |  |  |  | 1.3 | [0.7;2.3] |  |  |  |  |  |
| **Performance Status**  **at screening** |  |  |  | 0.10 |  |  |  |  |  | 0.03 |  |  |  |  |
| PS 0 | 63 | 1.0 |  |  |  |  |  | 1.0 |  |  |  |  |  |  |
| PS 1-2 | 79 | 1.4 | [0.9;1.9] |  |  |  |  | 1.6 | [1.1;2.4] |  |  |  |  |  |
